# Microbiota-mediated disease resistance in plants

**DOI:** 10.1371/journal.ppat.1007740

**Published:** 2019-06-13

**Authors:** Nathan Vannier, Matthew Agler, Stéphane Hacquard

**Affiliations:** 1 Max Planck Institute for Plant Breeding Research, Cologne, Germany; 2 Institute of Microbiology, Friedrich Schiller University, Jena, Germany; THE SAINSBURY LABORATORY, UNITED KINGDOM

## Introduction

Plant pathogens represent a constant and major threat to global food production, with 20%–30% global crop losses estimated, principally in food-deficit areas [1]. Pesticide use, breeding of resistance genes, and genetic manipulation of plant immune components have helped to mitigate this threat. However, rapid evolution of pathogen resistance and virulence, together with host range expansion and host jumps, contribute to severe disease outbreaks, especially in the context of current agricultural practices [2]. This underscores the need to reduce the lag time between the appearance of new diseases and development of protective measures effective on a broad range of pathogens and host plants.

In this context, microbial products and inoculants for plant protection have recently gained attention thanks to the large efforts made to systematically isolate, identify, and characterize plant-associated microbes that engage in intimate association with healthy plants [3]. Recent findings indicate that individual- and community-level features provided by plant microbiota members can confer extended immune functions to the plant host. Importantly, the traits lent by “beneficial” microbes strongly depend on the interplay between the soil nutrient status and the plant immune system [4, 5]. Thus, the successful implementation of microbiota-mediated disease protection will depend on our mechanistic understanding of how microorganisms interact with their hosts and with one another in natural environments. Here, we seek to synthesize the state of this cross-disciplinary field to bridge the gap toward rational design of synthetic microbial communities (SynComs) [6] with broad, durable, and flexible plant protective activities.

## Microbiota-modulated immunity (MMI) enhances disease resistance

**Fig 1:**
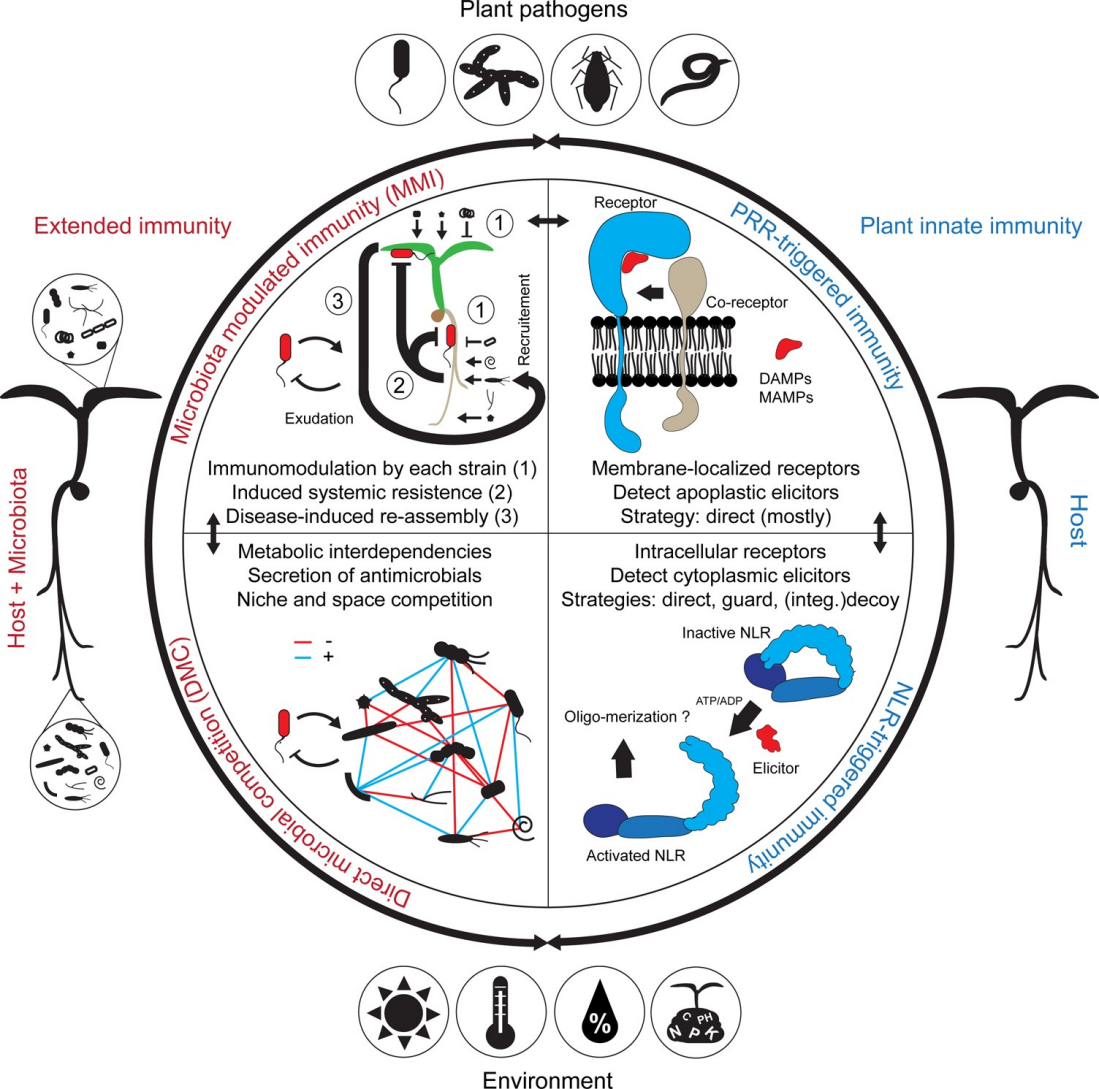
Microbiota-mediated extension of the plant immune system. Extracellular PRRs recognize MAMPs and DAMPs at the cell membrane. Intracellularly, NLRs recognize pathogen effectors either directly or indirectly by monitoring host proteins targeted by effectors. This innate immune system is modulated by the microbiota (i.e., MMI), which induces systemic resistance and enhances plant resistance to pathogens. In addition, the plant microbiota provides direct protective activity against microbial pathogens via DMC. DMC includes competition for nutrients and space as well as secretion of antimicrobials, and these interactions are integrated into a complex network that dictates pathogen growth *in planta*. ADP, adenosine diphosphate; ATP, adenosine triphosphate; DAMP, damage-associated molecular pattern; DMC, direct microbial competition; NLR, nucleotide-binding domain and leucine-rich repeat-containing receptor; MAMP, microbe-associated molecular pattern; MMI, microbiota-modulated immunity; PRR, pattern recognition receptor.

Land plants have evolved a complex innate immune system comprising membrane-localized receptors (pattern recognition receptors [PRRs]) and intracellular receptors (nucleotide-binding domain and leucine-rich repeat-containing receptors [NLRs]) that detect apoplastic and cytoplasmic elicitors, respectively, and activate immune outputs against microbial pathogens (**Fig 1**). Although some studies identified commensals that are able to evade PRR-triggered immunity, the conserved nature of microbe-associated molecular patterns (MAMPs) suggests that PRRs cannot efficiently discriminate commensal from pathogenic microbes [7]. Because healthy plants in nature are extensively colonized by these commensals, it is conceivable that the immune system and the microbiota may instruct each other [7] beyond the simple coevolutionary arms race between plants and pathogens. Consistent with this observation, recent evidence indicates that bacterial commensals can activate or suppress plant immune responses [8, 9] and that an intact immune system is required for the accommodation of beneficial microbes [10, 11].

Root microbiota–mediated stimulation of plant innate immunity has been extensively described to confer resistance against various microbial leaf pathogens (a phenomenon referred to as priming or induced systemic resistance [ISR]) [12, 13]. ISR has been well described in *Arabidopsis thaliana*, and the identified mechanisms controlling its onset appear to be conserved for different organisms. Particularly, the transcription factor MYB72 plays a key role in the regulation of ISR triggered by *Trichoderma* spp. fungi and the bacterium *Pseudomonas simiae* [14, 15]. Interestingly, MYB72 is also involved in *A*. *thaliana’s* response to iron deficiency [16], suggesting a direct interplay between nutrient stress and immunity. ISR may occur because plants have evolved to use microbial molecules as developmental signals for plant immune system maturation [17], implying that early contact with microbe-derived molecules is needed for plant survival in natural soils. Recent studies indicate that *A*. *thaliana* leaf infection by the biotrophic pathogens *Pseudomonas syringae* and *Hyaloperonospora arabidopsidis* results in root recruitment of beneficial ISR-inducing bacteria [18, 19], mediated by modification of root exudation profiles [18]. Remarkably, the presence of ISR-inducing strains can further drive root secretion of antimicrobial coumarin compounds that shape the root microbiota and mobilize potentially beneficial bacteria, including ISR-inducing strains [20]. This suggests a self-reinforcing immunity and recruitment loop, which constitutes a promising tool to manipulate beneficial microbiota functions for stable plant protection across generations. Interestingly, the same exudates that shape root microbiota often function in abiotic stress responses [21, 22], reflecting that root recruitment systems—similar to the plant immune system itself—have evolved multiple functions.

## Direct microbial competition (DMC) supplements plant innate immunity

Direct pathogen suppression by microbiota members has been repeatedly reported in plant roots [23–26] and leaves [27, 28]. These interactions are highly diverse and include secretion of antimicrobial compounds [28, 29], hyperparasitism [30], and competition for resources like nutrients or space [31], which ultimately mitigate pathogen growth (**Fig 1**). Recently, it has been shown that although roots of *A*. *thaliana* are colonized by deleterious filamentous eukaryotes in natural populations, plants remain healthy because of the presence of coresident bacteria that maintain fungal balance in plant roots and promote host survival [32]. This raises the possibility that, in nature, plants may equally rely on their resident bacteria and immune system to restrict pathogen invasion. In tomato, microbiota structure and composition are predictors of disease resistance, but trophic network architecture, which describes how resident microbiota and pathogens interact with each other in terms of resource usage, was equally important [33]. At the community level, DMC constitutes a complex network of interactions that determines microbes’ coexistence and thereby ultimately dictates pathogen invasion in plant tissues (**Fig 1**). Thus, it is becoming increasingly clear that potential applications for plant protection depend on the understanding and manipulation of multispecies and multikingdom interactions that are critical for microbiota-mediated disease resistance.

## Rationally designed SynComs can provide robust plant protection

**Fig 2:**
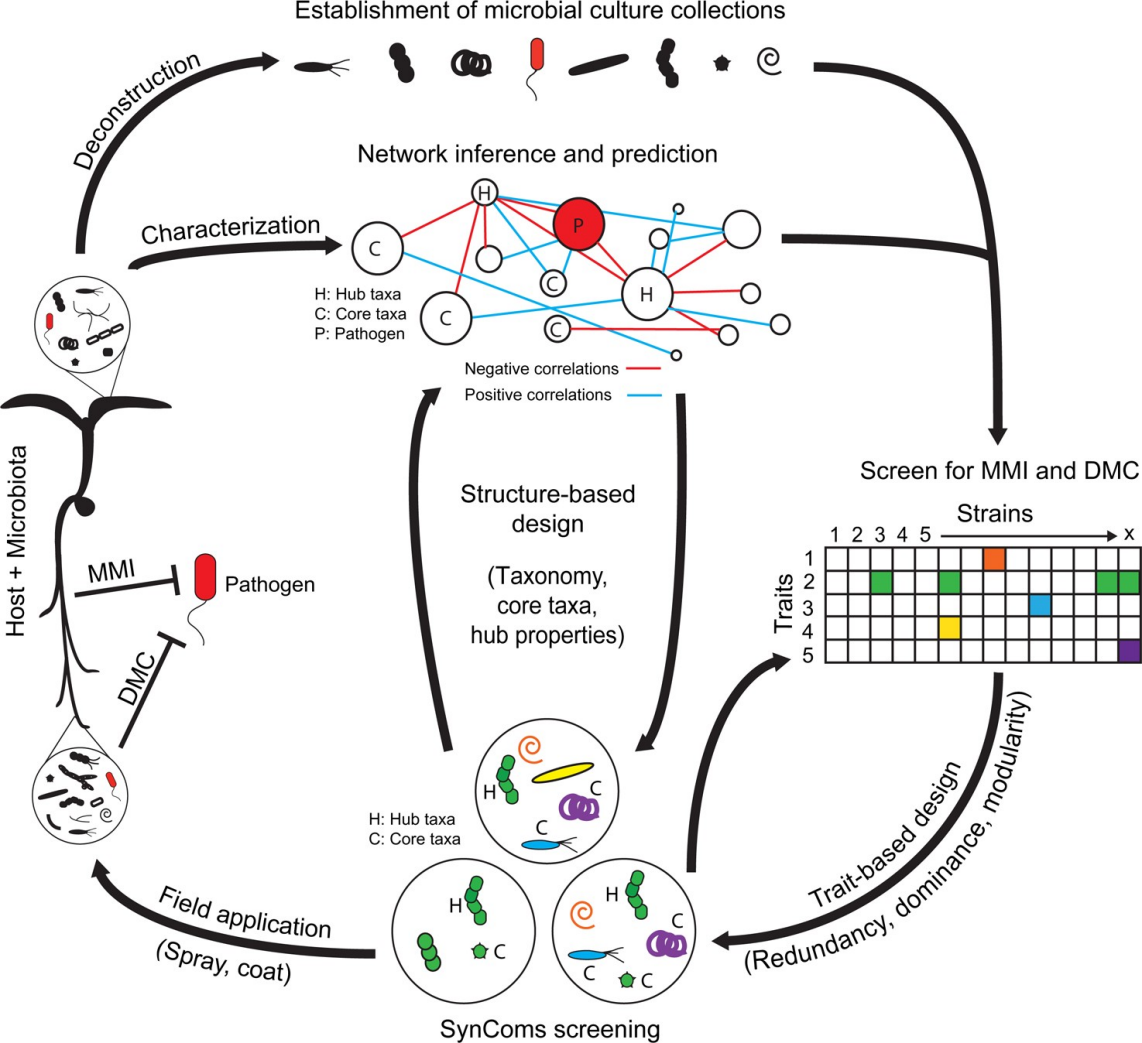
Rational design of SynComs with predictable pathogen biocontrol activities. Starting in the field where a pathogen outbreak has occurred, the microbiota of diseased and healthy plants are characterized and isolated. The isolates are screened in binary microbe–microbe and *in planta* ternary interactions to detect and catalogue traits linked to DMC and MMI. From the obtained catalogue, activities of individual strains can be used to design more complex SynComs while taking into account trait redundancy, dominance, and modularity. In parallel, interaction networks are inferred from sequencing data, and potential key organisms are identified based on hub structural properties or functional modules. This network inference helps with prioritizing candidate strains for targeted screening of DMC and MMI traits. Both trait-based and structure-based approaches can inform the rational design of SynComs with stable and effective biocontrol activities in the field. DMC, direct microbial competition; MMI, microbiota-modulated immunity; SynComs, synthetic microbial communities.

Single strains carrying specific functions under laboratory conditions have already been used for their biocontrol activities under field conditions, but they provide beneficial activities only in some cases [3]. This is due to the fact that microbiota-mediated disease resistance occurs at the interface between plants and their local environments, where plant-associated communities of prokaryotic and eukaryotic microbes are hyperdiverse and changing over time. Therefore, how can microbiota-encoded traits like MMI or DMC be successfully maintained in the field to increase crop resilience? Several examples suggest that defined SynComs could confer more efficient plant protection than individual strains [24, 32] and argue for the use of native, locally adapted plant-associated microbes for efficient plant protection in the field [24, 32, 34]. The recent demonstration that most abundant plant-associated microbes can be isolated and maintained axenically [32, 35] opens new avenues to systematically screen for desirable traits using microfluidic systems, high-throughput screens, or microbiota reconstitution experiments with SynComs and germ-free plants (**Fig 2**). Thus, the design of SynComs based on traits involved in MMI and/or DMC represents a promising direction to achieve robust plant protective activities against pathogens. Here, we illustrate two emerging tools and concepts that could lead toward a more targeted screening approach and to higher SynCom success in the field (**Fig 2**).

### The robustness of beneficial microbial trait expression is increased by redundancy and dominance

In order to design SynComs with predictable beneficial outcomes for plant health in the field, understanding whether selected traits are retained in a community context is essential. Specifically, traits that are redundant (i.e., expressed by multiple strains) and dominant (i.e., expressed whenever one strain has the respective function) across SynCom members are likely to lead to robust expression of the trait in a community context (**Fig 2**). In plants, several bacterial root commensals belonging to the families Comamonadaceae and Pseudomonadaceae were able to protect *A*. *thaliana* against root-derived filamentous eukaryotes, illustrating how one functional trait can be expressed by phylogenetically distant bacteria [32]. Because deployed SynComs will be challenged by many environmental microorganisms, redundant traits also need to be dominantly expressed in multiple contexts. Garrido-Oter and colleagues (2018) [8] observed that root growth promotion and MAMP response suppression by Rhizobiales root commensals are dominant traits, the corresponding functions being expressed regardless of the presence of another bacterial cocolonizer. These advances lend additional support to the idea that traits observed in monoassociation with the host can at least partially inform the design of SynComs with predictable outcomes, as was recently reported [36]. However, even if the observed root growth promotion and MAMP response suppression phenotypes observed in monoinoculation often predict dual-inoculation outcomes, some combinations can result in synergistic effects [8]. Importantly, the emergence of such new properties is a phenomenon that cannot be predicted from trait screening in binary interactions but that can be tested experimentally in a SynCom context (**Fig 2**).

### Network analysis as a tool for the design of stable and predictable SynComs

Screening for MMI and DMC traits can be a highly time-consuming approach considering the very high diversity of microbes that can be isolated from plants. Analytic tools have been recently developed that can be used to target such screening and to improve the likelihood of SynCom success in the field. A good example is the significant progress made in ecological network inference. In short, inferred networks break down complex ecological systems into “interactions” (usually correlations) between measured entities, revealing, for example, how microbial taxa, genes, and abiotic factors are linked to host phenotypes. Analyzing these networks guides the formation of testable hypotheses about which (groups of) entities drive phenotypes ahead of lab screening (**Fig 2**). For example, this approach was used to identify potential bacterial antagonists of oak powdery mildew by characterizing plants between their healthy state and during disease progression [37]. Inferred networks of microbial interactions have also been used to reveal functionally related rhizosphere microorganisms [38] and keystone species in microbial assemblies that influence community structures [39]. Given the influence of keystone species, this approach can be used to identify possible points of control to manipulate microbial diversity and to characterize strains based on their persistence in the field. In summary, network inference provides the opportunity to take into account the local context by linking microbial and abiotic factors to phenotypes, providing a pathway to maximize SynCom persistence and trait expression success in the field [39].

## Concluding remarks

It is now critical to further develop bottom-up experimental approaches to improve our understanding of dominance and modularity of desirable traits within SynComs. Cataloguing such knowledge on microbial traits and their behavior in a community context will allow the establishment of a microbial toolbox for microbiota-mediated pathogen protection and the rational design of SynComs with modular functions related to MMI and DMC. In parallel or in combination with this cataloguing, microbial network inference will play a key role in identifying candidate biocontrol taxa and in the design of SynComs with stable and predictable outcome in the field. In addition to the mentioned benefits for direct application in the field, this approach is likely to provide insights into the following unresolved fundamental questions: (1) Can we detect phylogenetic signals for MMI and DMC traits that could be used to predict beneficial outcomes for the plant host? (2) Do immunity-modulating microbes act through similar or different immune pathways? And (3) as a consequence of the previous question, do the identified MMI and DMC traits act independently, synergistically, or antagonistically?
